# A strong host response and lack of MYC expression are characteristic for diffuse large B cell lymphoma transformed from nodular lymphocyte predominant Hodgkin lymphoma

**DOI:** 10.18632/oncotarget.12363

**Published:** 2016-09-30

**Authors:** Bianca Schuhmacher, Benjamin Rengstl, Claudia Döring, Julia Bein, Sebastian Newrzela, Uta Brunnberg, Hans Michael Kvasnicka, Martine Vornanen, Ralf Küppers, Martin-Leo Hansmann, Sylvia Hartmann

**Affiliations:** ^1^ Dr. Senckenberg Institute of Pathology, Goethe University, Frankfurt am Main, Germany; ^2^ Department of Internal Medicine 2, Hospital of the J. W. Goethe University, Frankfurt am Main, Germany; ^3^ Department of Pathology, Tampere University Hospital and University of Tampere, Tampere, Finland; ^4^ Institute of Cell Biology (Cancer Research), Faculty of Medicine, University of Duisburg-Essen, Essen, Germany

**Keywords:** nodular lymphocyte predominant Hodgkin lymphoma, diffuse large B cell lymphoma, T cell/histiocyte rich large B cell lymphoma, host response, gene expression profiling

## Abstract

Nodular lymphocyte predominant Hodgkin lymphoma (NLPHL) is an indolent lymphoma, but can transform into diffuse large B cell lymphoma (DLBCL), showing a more aggressive clinical behavior. Little is known about these cases on the molecular level. Therefore, the aim of the present study was to characterize DLBCL transformed from NLPHL (LP-DLBCL) by gene expression profiling (GEP). GEP revealed an inflammatory signature pinpointing to a specific host response. In a coculture model resembling this host response, DEV tumor cells showed an impaired growth behavior. Mechanisms involved in the reduced tumor cell proliferation included a downregulation of *MYC* and its target genes. Lack of MYC expression was also confirmed in 12/16 LP-DLBCL by immunohistochemistry. Furthermore, CD274/PD-L1 was upregulated in DEV tumor cells after coculture with T cells or monocytes and its expression was validated in 12/19 cases of LP-DLBCL. Thereby, our data provide new insights into the pathogenesis of LP-DLBCL and an explanation for the relatively low tumor cell content. Moreover, the findings suggest that treatment of these patients with immune checkpoint inhibitors may enhance an already ongoing host response in these patients.

## INTRODUCTION

Diffuse large B cell lymphoma (DLBCL) is the most frequent type of aggressive B cell lymphoma and comprises a heterogeneous mixture of cases [1]. DLBCL can be subdivided according to its cell of origin into germinal center B cell (GCB)-like and activated B cell (ABC)-like lymphomas [2, 3]. Assignment to GCB and ABC categories has also been associated with prognosis [3]. Currently, new treatment options for ABC-DLBCL are evolving, since these lymphomas are dependent on chronic active B cell receptor signaling and frequently engage self-antigens [4]. Some immunohistochemical classifiers discriminate between DLBCL of GCB and Non-GCB type [5, 6].

Nodular lymphocyte predominant Hodgkin lymphoma (NLPHL) is a subtype of Hodgkin lymphoma (HL). In contrast to classical HL it has several particular features, which include a preserved B cell phenotype of the tumor cells [7], the LP cells, and a higher frequency in males as well as a higher risk of transformation into DLBCL. We previously showed in two composite lymphoma cases with available frozen tissue the clonal relatedness of the NLPHL and DLBCL component [8]. These DLBCL transformed from NLPHL (LP-DLBCL) often have a sheet-like growth pattern [9]. However, among the immunohistochemical markers tested, none of them was highly specific for LP-DLBCL [9]. The clinical behavior of LP-DLBCL is controversially discussed at the moment. There are data indicating a more aggressive course [10], but most studies showed a favorable outcome compared to conventional DLBCL [11–15]. Therefore, it would be desirable to recognize these cases as transformed NLPHL, even when only the DLBCL part is biopsied. Recently, we demonstrated that mechanisms of transformation from NLPHL into LP-DLBCL can be heterogeneous and that in some cases few additional mutations may be sufficient for transformation, whereas in other cases a huge mutational load together with complex chromothripsis-like genomic aberrations are present [8]. The aim of the present study was to characterize LP-DLBCL by gene expression profiling (GEP) to gain further insight into its pathogenesis.

## RESULTS

### Gene expression analysis reveals a strong host response signature in LP-DLBCL

**Figure 1:**
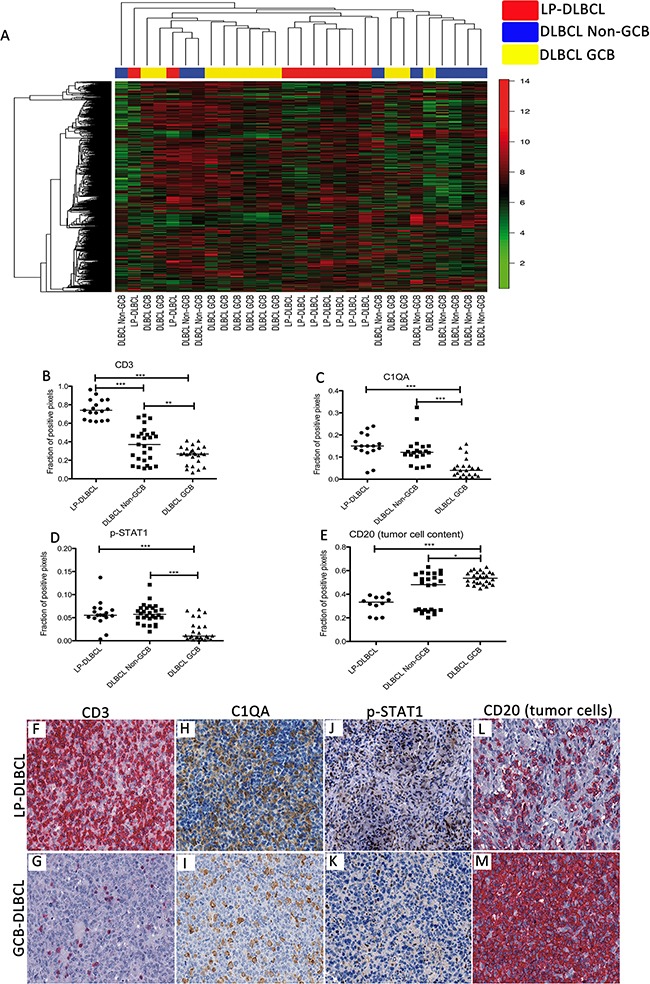
Gene expression profiling recognizes LP-DLBCL as a core group among all DLBCL **A.** Unsupervised hierarchical clustering of gene expression data of LP-DLBCL (red), GCB type DLBCL (yellow) and Non-GCB type DLBCL (blue). We considered 1203 probe sets with a standard deviation > 2. **B.** Quantification of the fraction of CD3-positive pixels reveals significantly higher numbers in LP-DLBCL compared with Non-GCB and GCB DLBCL (***p<0.0001, **p=0.0093 paired t-test). **C.** Quantification of the fraction of C1QA-positive pixels reveals significantly higher numbers in LP-DLBCL compared with GCB DLBCL (***p<0.0001, Mann-Whitney-test). **D.** Quantification of the fraction of pSTAT1-positive pixels reveals significantly higher numbers in LP-DLBCL compared with GCB DLBCL (***p≤0.0003, Mann-Whitney-test). **E.** The fraction of CD20-positive pixels was significantly lower in LP-DLBCL compared with GCB type DLBCL (***p<0.0001, * p=0.0221, Mann-Whitney-test). **F.** Representative example of CD3 immunostaining of a LP-DLBCL (200x). **G.** Representative example of CD3 immunostaining of a GCB type DLBCL (200x). **H.** Representative example of C1QA immunostaining of a LP-DLBCL (200x). **I.** Representative example of C1QA immunostaining of a GCB type DLBCL (200x). **J.** Representative example of pSTAT1 immunostaining of a LP-DLBCL (200x). K. Representative example of pSTAT1 immunostaining of a GCB type DLBCL (200x). **L.** Representative example of CD20 immunostaining of a LP-DLBCL (200x). **M.** Representative example of CD20 immunostaining of a GCB type DLBCL (200x).

Gene expression profiling (GEP) of whole sections from 9 formalin-fixed, paraffin-embedded LP-DLBCL as well as 20 conventional DLBCL was performed. Clinical data of the cases investigated by GEP are listed in Supplementary Table S1. Unsupervised hierarchical clustering of all 20 conventional DLBCL (11 GCB and 9 Non-GCB type) and 9 LP-DLBCL cases revealed a core group of 7 LP-DLBCL cases, which clustered together. All other cases arranged around this core group, without clear separation of GCB- or Non-GCB-DLBCL (Figure 1A).

A supervised comparison of LP-DLBCL against all other DLBCL cases revealed an overexpression of T cell- and macrophage-associated genes (GZMK, MT1H, C1QA, CD2, STAT1, CD3D, CD3G, SOD2, Table 1) in the LP-DLBCL. Granzyme K (GZMK) is expressed in cytotoxic T cells, therefore possibly reflecting a host anti-lymphoma response. To validate these results, the fraction of positive pixels was assessed in immunostained sections, in order to automatically quantify the T cell antigen CD3 and the macrophage antigens C1QA and p-STAT1, which were identified to be differentially expressed. Indeed, CD3 immunostaining revealed significantly more positive pixels in LP-DLBCL cases when compared with GCB type DLBCL and Non-GCB type DLBCL, respectively (p<0.0001, t-test, Figure 1). Both, C1QA and p-STAT1, usually expressed in macrophages, showed higher expression values in LP-DLBCL compared with GCB type DLBCL (Figure 1, p<0.0001 and p=0.0003, respectively, Mann-Whitney-Test). Despite the slightly higher number of C1QA-positive pixels in LP-DLBCL compared with Non-GCB type DLBCL, the expression of C1QA and p-STAT1 was not significantly different in LP-DLBCL when compared with Non-GCB type DLBCL.

**Table 1:** Top 25 genes up regulated in LP-DLBCL compared with conventional DLBCL

Fold change	p-value	FDR	Gene Symbol	Gene Description
4.8	0.0014	0.0331	*GZMK*	granzyme K (granzyme 3; tryptase II)
3.4	0.0007	0.0251	*GBP1*	guanylate binding protein 1, interferon-inducible
3.4	0.0012	0.0322	*SMG1*	SMG1 phosphatidylinositol 3-kinase-related kinase
3.1	0.0014	0.0331	*MT1H*	metallothionein 1H
3.0	0.0021	0.0409	*GBP5*	guanylate binding protein 5
3.0	0.0001	0.0086	*SLAMF7*	SLAM family member 7
2.9	0.0004	0.0180	*C1QA*	complement component 1, q subcomponent, A chain
2.8	0.0015	0.0331	*PYHIN1*	pyrin and HIN domain family, member 1
2.8	0.0002	0.0143	*GIMAP4*	GTPase, IMAP family member 4
2.7	0.0008	0.0265	*CD2*	CD2 molecule
2.6	0.0010	0.0294	*GBP2*	guanylate binding protein 2, interferon-inducible
2.6	0.0009	0.0278	*LCP2*	lymphocyte cytosolic protein 2 (SH2 domain containing leukocyte protein of 76kDa)
2.6	0.0046	0.0620	*SCARNA8*	small Cajal body-specific RNA 8
2.5	0.0003	0.0179	*WARS*	tryptophanyl-tRNA synthetase
2.5	0.0000	0.0086	*STOM*	stomatin
2.5	0.0014	0.0331	*SCARNA6*	small Cajal body-specific RNA 6
2.5	0.0012	0.0322	*STAT1*	signal transducer and activator of transcription 1, 91kDa
2.4	0.0005	0.0227	*SCARNA10*	small Cajal body-specific RNA 10
2.4	0.0000	0.0086	*FYN*	FYN proto-oncogene, Src family tyrosine kinase
2.4	0.0007	0.0251	*SNX10*	sorting nexin 10
2.3	0.0092	0.0937	*CD3D*	CD3d molecule, delta (CD3-TCR complex)
2.3	0.0039	0.0574	*CD3G*	CD3g molecule, gamma (CD3-TCR complex)
2.3	0.0073	0.0815	*SOD2*	superoxide dismutase 2, mitochondrial
2.3	0.0003	0.0179	*RNF213*	ring finger protein 213
2.3	0.0001	0.0101	*CST7*	cystatin F (leukocystatin)

FDR, false discovery rate

Several genes known to be expressed in B cells were expressed at lower levels in LP-DLBCL than in conventional DLBCL, including MS4A1 (CD20), CD24 [16], TCL1A [17] and BLNK [18] (Table 2). Downregulation of these B cell-specific transcripts indicates either a reduced tumor cell content or a partially downregulated B cell phenotype in the tumor cells. Since reactive B cells rarely occur among the non-neoplastic bystander cells, the fraction of CD20-positive pixels reflects the tumor cell content, and was quantified. It was significantly lower in LP-DLBCL compared with GCB-DLBCL (Figure 1E, 1L, 1M, p<0.0001, Mann-Whitney-Test). However, the average intensities of CD20-immunostaining did not differ between LP-DLBCL and GCB-DLBCL (data not shown), arguing against a downregulated B cell phenotype in the tumor cells of LP-DLBCL.

**Table 2:** Top 15 genes down regulated in LP-DLBCL compared with conventional DLBCL

Fold change	p-value	FDR	Gene Symbol	Gene Description
3.3	0.0012	0.0322	*CD24*	CD24 molecule
3.0	0.0031	0.0519	*MT-TL2*	mitochondrially encoded tRNA leucine 2 (CUN)
2.9	0.0034	0.0529	*RGS13*	regulator of G-protein signaling 13
2.8	0.0002	0.0161	*RPLP0*	ribosomal protein, large, P0
2.7	0.0029	0.0500	*MS4A1*	membrane-spanning 4-domains, subfamily A, member 1
2.6	0.0000	0.0086	*MT-TP*	mitochondrially encoded tRNA proline
2.4	0.0037	0.0553	*TCL1A*	T-cell leukemia/lymphoma 1A
2.4	0.0011	0.0318	*BLNK*	B-cell linker
2.3	0.0000	0.0086	*RPL23A*	ribosomal protein L23a
2.3	0.0019	0.0387	*USMG5*	up-regulated during skeletal muscle growth 5 homolog (mouse)
2.3	0.0003	0.0179	*RPL12P1*	ribosomal protein L12 pseudogene 1
2.2	0.0069	0.0780	*C12orf75*	chromosome 12 open reading frame 75
2.1	0.0005	0.0228	*RPL21*	ribosomal protein L21
2.1	0.0003	0.0180	*EIF2S3*	eukaryotic translation initiation factor 2, subunit 3 gamma, 52kDa
2.1	0.0059	0.0700	*MT-TM*	mitochondrially encoded tRNA methionine

FDR, false discovery rate

**Figure 2:**
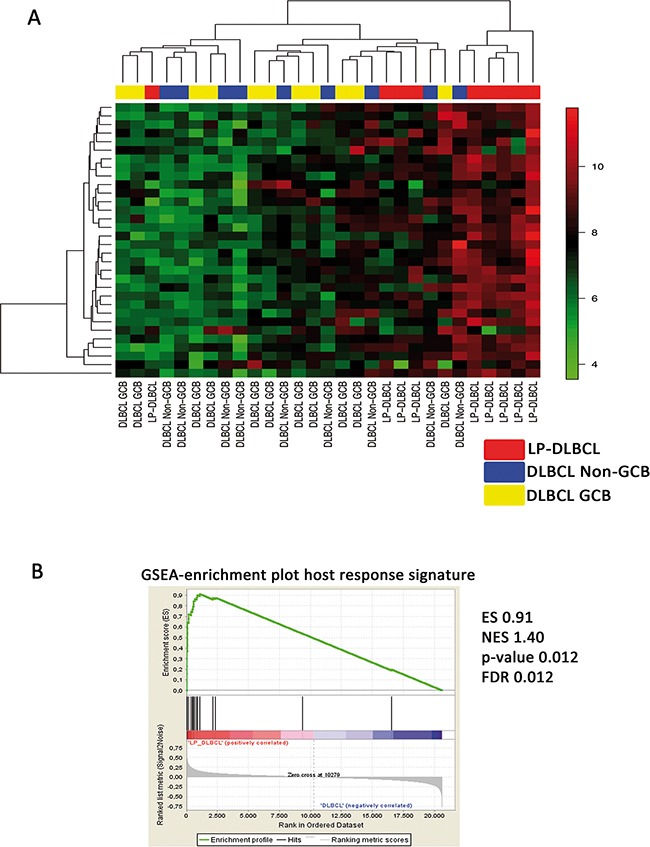
LP-DLBCL is characterized by a strong inflammatory infiltrate **A.** Supervised clustering based on the host inflammatory response signature identified in THRLBCL [19]. **B.** Gene set enrichment analysis shows a significant enrichment of the host inflammatory response signature [19] in LP-DLBCL compared with all other DLBCL. ES enrichment score, NES normalized enrichment score, FDR false discovery rate.

Since a prominent, partly cytotoxic host response in LP-DLBCL was identified, we compared our GEP data with a previous study by Monti et al. [19], in which a subset of DLBCL with a strong host inflammatory response was detected. When this host response signature was applied to the present data set, eight of nine LP-DLBCL clustered together (Figure 2A) and showed a significant enrichment of this signature using gene set enrichment analysis (p=0.0120, Figure 2B), confirming the validity of the present data even across different array platforms.

### The inflammatory infiltrate in LP-DLBCL shows a low CD4/CD8 ratio and a high content of macrophages

**Figure 3:**
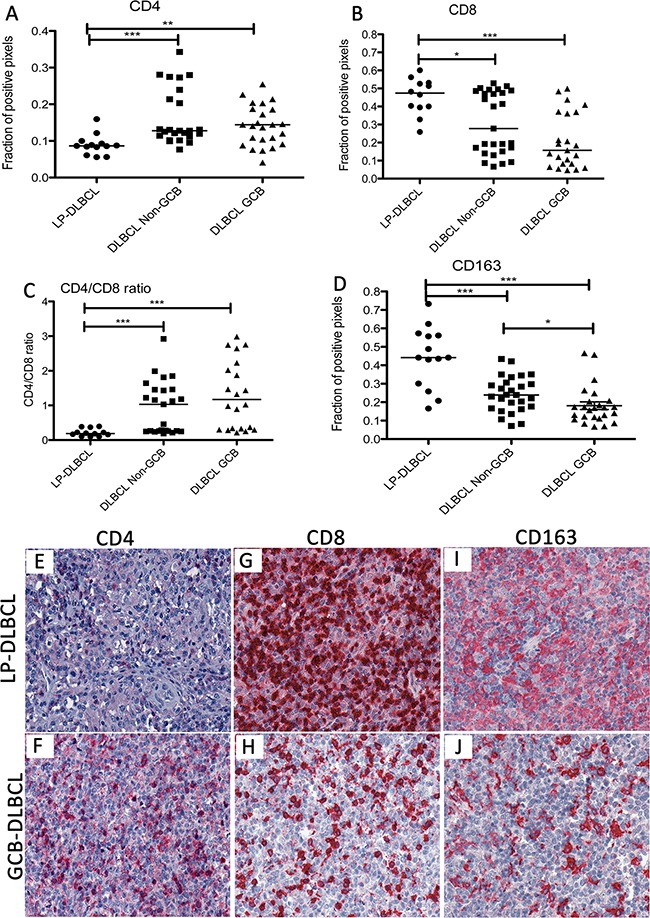
The inflammatory infiltrate in LP-DLBCL shows a low CD4/CD8 ratio and a high content of macrophages **A.** The fraction of CD4-positive pixels was significantly lower in LP-DLBCL compared with Non-GCB and GCB type DLBCL (***p<0.0001, **p=0.0035, Mann-Whitney-test). **B.** Quantification of the fraction of CD8-positive pixels reveals significantly higher numbers in LP-DLBCL compared with Non-GCB and GCB DLBCL (***p=0.0002, *p=0.0169, Mann-Whitney-test). **C.** The CD4/CD8 ratio is significantly decreased in the microenvironment of LP-DLBCL compared with Non-GCB and GCB DLBCL (***p≤0.0003, Mann-Whitney-test). **D.** Quantification of the fraction of CD163-positive pixels reveals significantly higher numbers in LP-DLBCL compared with Non-GCB and GCB DLBCL (***p≤0.0003, *p=0.0109, Mann-Whitney-test). **E.** Representative example of LP-DLBCL in CD4 immunostaining (200x). **F.** Representative example of a GCB type DLBCL in CD4 immunostaining (200x). **G.** Representative example of LP-DLBCL in CD8 immunostaining (200x). H. Representative example of a GCB type DLBCL in CD8 immunostaining (200x). **I.** Representative example of LP-DLBCL in CD163 immunostaining (200x). **J.** Representative example of a GCB type DLBCL in CD163 immunostaining (200x).

Since we identified in LP-DLBCL mainly transcripts of reactive bystander cells as being differentially expressed in comparison to conventional DLBCL, numbers of CD4-positive and CD8-positive T cells as well as CD163-positive macrophages were quantified by the Aperio positive pixel count algorithm in these groups (Figure 3). Surprisingly, in CD4 immunostaining, a significantly lower number of positive pixels was detected in LP-DLBCL when compared with DLBCL of the GCB and Non-GCB types (Figure 3A, E, F, p=0.0035 and p<0.0001, respectively, Mann-Whitney-Test). In contrast, LP-DLBCL showed significantly higher numbers of CD8-positive pixels when compared with both GCB and Non-GCB type DLBCL (Figure 3B, 3G, 3H p=0.0002 and p=0.0169, respectively, Mann-Whitney-Test). Also the CD4/CD8 ratio was significantly decreased in LP-DLBCL compared with GCB and Non-GCB type DLBCL (Figure 3C, p=0.0001 and p=0.0003, respectively, Mann-Whitney-Test). CD163 immunostaining confirmed a significantly higher amount of macrophages in LP-DLBCL compared to GCB and Non-GCB type DLBCL (Figure 3D, 3I, 3J p<0.0001 and p=0.0003, respectively, Mann-Whitney-Test).

### Host response in vitro reveals a MYC downregulation in the tumor cells

**Figure 4:**
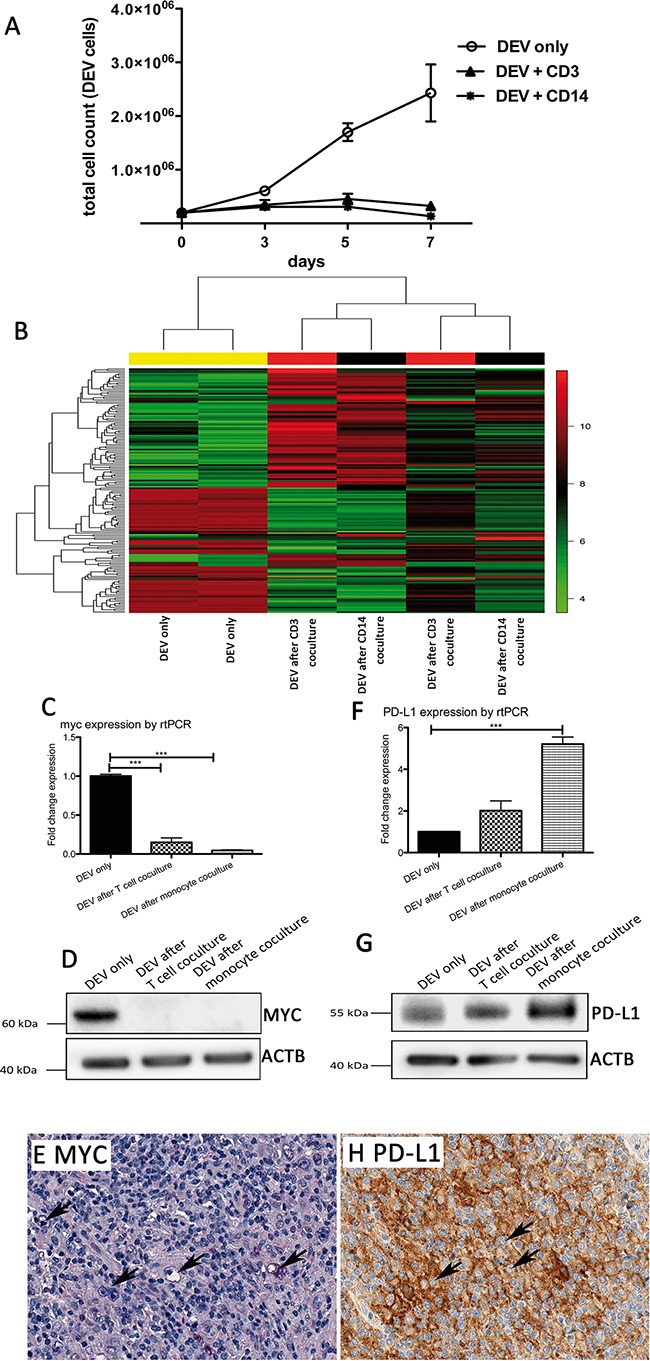
Growth of the NLPHL cell line DEV is impaired in the presence of T cells or monocytes **A.** Growth curves of the NLPHL cell line DEV in coculture with T cells or monocytes compared to a corresponding monoculture. **B.** Unsupervised GEP clustering of DEV cells in monoculture and DEV cells isolated after 5 days from coculture with T cells or monocytes. Two representative replicates of several experiments were analyzed for changes in GEP. We considered 158 probe sets with a standard deviation > 2 for the cluster analysis. **C.** *MYC* mRNA expression determined by Taqman realtime RT-PCR in DEV cells after coculture with T cells or monocytes, relative to GAPDH and relative to DEV cells from monoculture (***p<0.0001, paired t-test). D. Western blot of MYC protein in representative samples of DEV cells after coculture with T cells or monocytes compared to a corresponding monoculture. ACTB was used as loading control. **E.** Example of an LP-DLBCL with lack of MYC expression in the majority of the tumor cells (200x). **F.** *CD274/PD-L1* mRNA expression determined by Taqman realtime RT-PCR in DEV cells after coculture with T cells or monocytes, relative to GAPDH and relative to DEV cells from monoculture (***p<0.0001, paired t-test). **G.** Western blot of PD-L1 protein in representative samples of DEV cells after coculture with T cells or monocytes compared to a corresponding monoculture. ACTB was used as loading control. **H.** Example of an LP-DLBCL with membrane bound CD274/PD-L1 expression in the tumor cells (200x).

Since a strong host response signature was observed in GEP of LP-DLBCL, we aimed to simulate this situation in vitro by establishing a coculture model of lymphoma cells with T cell and monocyte fractions isolated from human peripheral blood mononuclear cells (PBMCs). Since no cell line representative for LP-DLBCL exists, the DEV cell line, which is the only available NLPHL cell line [20, 21], derived from a long standing NLPHL, was used. In order to avoid a major histocompatibility complex class I or class II (MHC-I or MHC-II)-mediated allogeneic anti-tumor reaction [22], DEV cells were tested for MHC expression. In line with previous studies [23, 24], neither MHC-I nor MHC-II expression was detected on DEV cells (Supplementary Figure S1). Therefore, MHC-unmatched PBMCs could be applied for coculture experiments. MHC expression on DEV cells was further monitored during coculture by flow cytometry, but remained negative (data not shown). The growth behavior of DEV cells was monitored over 7 days in monoculture or in coculture either with CD3-positive T cells or CD14-positive monocytes. Under both coculture conditions, DEV cells showed a growth arrest (Figure 4A). To further elucidate the mechanisms involved in growth suppression of DEV cells due to the presence of T cells and monocytes, GEP of DEV cells purified from 5 days-old cocultures was performed in comparison to corresponding monocultures. Interestingly, a coculture-induced switch in GEP was observed (Figure 4B). The most strongly upregulated genes after coculture with either T cells or monocytes were *HSN2*, controlling the transport of sodium and chloride ions [25], *RAB3A,* involved in exocytosis [26] and *FABP3*, regulating fatty acid metabolism [27] (Supplementary Table S2). In contrast, the most strongly downregulated transcripts were *MYC*, miR155 and *P2RY14* (Supplementary Table S3). Of note, DEV cells isolated from cocultures did not show an enhanced expression of T cell or monocyte transcripts, confirming a highly efficient depletion of blood cells prior to GEP (Supplementary Figures S2 and S3).

Gene set characterization using the Genomatix Pathway System revealed several significantly enriched pathways, most of them being negatively regulated (Table 3). Among the top enriched and negatively regulated pathways were the E2F transcription factor network, validated targets of the MYC transcription factor, the MYB transcription factor network as well as cyclins and cell cycle regulation. A negative regulation of these pathways [28] is consistent with the observed reduced proliferation of DEV cells under coculture conditions. Downregulation of MYC was also confirmed on transcript and protein level in DEV cells after coculture (Figure 4C and 4D) and in 12/16 primary LP-DLBCL with > 90% MYC-negative tumor cells (Figure 4E). Surprisingly, there was no general enrichment of pro-apoptotic genes in the pathway analysis (nor in a heat map of pro-apoptotic genes, Supplementary Figure S4). In contrast, DEV cells showed a 2.4-fold upregulation of *IL23A* after coculture experiments, potentially explaining the reduced proliferative capacity of DEV cells, since IL23 was shown to inhibit cell proliferation of lymphoblastic leukemia cell lines in vitro [29].

**Table 3:** Top ten enriched canonical signaling pathways according to Genomatix Pathway System in DEV cells after coculture with T cells/monocytes

Pathway	p-value	Upregulated genes	Downregulated genes
E2F transcription factor network	0.0005	*CDK2, CDKN1A*	*MYC, CCND3, MCM3, PRMT5, POLA1*
Validated targets of MYC transcription factor	0.0046	*-*	*MYC, LDHA, NME1, RUVBL1, EIF4G1, DKC1*
MYB transcription factor network	0.0059	*CDKN1A*	*MYC, MAT2A, SIN3A, MYB, HSPA8*
Cyclins and cell cycle regulation	0.0093	*CDK2, CDKN1A*	*CCND3*
Cyclin E destruction pathways	0.0121	*CDK2*	*CUL1*
ID	0.0131	*CDK2, ID3*	*ELK3*
IL2-beta chain in T cell activation	0.0141	*IL2RB*	*MYC, NMI, CCND3*
CD28 signalling	0.0141	*MAPK8, HLA-DRA, HLA-DQB1*	*CD86*
E2F1 destruction pathway	0.0150	*CDK2*	*CUL1*
IL2 signaling events mediated by STAT5	0.0160	*IL2RB*	*MYC, CCND3*

### Inhibition of anti-lymphoma host response activation by PD-L1

Although we could not monitor an enhanced expression of MHC surface molecules on DEV cells by flow cytometry during the coculture experiments, MHC class II transcripts were upregulated in GEP (HLA-DRA 2.3- and HLA-DQB1 2.5-fold), pointing to a possible interaction of DEV cells with T cells. Interestingly, DEV cells also presented a 2.1-fold upregulation of CD274/PD-L1 after coculture experiments suggesting an inhibition of T cell activation in the coculture. A slight upregulation of CD274/PD-L1 was also confirmed on transcript (Figure 4F) and protein level (Figure 4G). To further investigate if this represents a general finding in LP-DLBCL, we tested 19 LP-DLBCL cases for PD-L1 expression by immunohistochemistry and observed in 12 cases (63%) a membrane bound expression in the tumor cells (Figure 4H). Therefore, PD-L1 was significantly more frequently expressed in LP-DLBCL than in DLBCL of the GCB type (2/22 cases) and of the Non-GCB type (3/26 cases, p<0.0001, Chi-Square-Test). In the PD-L1-negative cases, PD-L1 expression was mainly restricted to histiocytes in the microenvironment.

## DISCUSSION

In the present study, GEP of a series of LP-DLBCL was performed and revealed a strong inflammatory host response, dominated by macrophages and cytotoxic T cells. A similar observation was made in a previous study by Monti *et al.* [19], when a large cohort of DLBCL not otherwise specified was investigated by GEP and a group with a prominent host response reaction was identified. In this group, particularly cases with features of T cell/histiocyte rich large B cell lymphoma (THRLBCL) were included. THRLBCL has previously been shown to have a large overlap with NLPHL [30–33], but usually presents with a more aggressive behavior than typical NLPHL, like LP-DLBCL. Although Monti et al. investigated a completely different case series and also the array platforms differed, seven of the top 25 genes overexpressed in LP-DLBCL cases in the present study, were also identified in the previous host response signature. Furthermore, some of the top 25 transcripts identified in LP-DLBCL, like *MT1H*, *C1QA*, *STAT1* and *SOD2*, were also shown to be expressed in the macrophages of THRLBCL [34, 35], suggesting a similar composition of the microenvironment in LP-DLBCL and THRLBCL. The strong overlap of microenvironment composition in LP-DLBCL and THRLBCL supports a close relationship between NLPHL, THRLBCL and LP-DLBCL, suggesting that THRLBCL might represent a transformation of NLPHL with reduced growth of the tumor cells and enhanced host response yielding a paucicellular pattern. Even in LP-DLBCL we could demonstrate a reduced tumor cell content compared with conventional DLBCL, however, not reaching below 10% as usually observed in THRLBCL [1].

A protective role of high numbers of CD8-positive T cells related to improved outcome was observed in DLBCL not otherwise specified [36]. It was furthermore observed that loss of HLA expression, B7 molecules and ICAM1 was associated with decreased infiltration of CD8-positive T cells in DLBCL [37]. And indeed, consistent with the high CD8 content in LP-DLBCL, 100% and 88% of cases were tested as positive for ICAM1 and HLA-DR expression by immunohistochemistry, respectively (data not shown). Likewise, THRLBCL has been described to have a relatively high CD8 content [38], which we also confirmed in a previous study [32]. THRLBCL may therefore represent the extreme form of LP-DLBCL with a maximum host response and slow tumor cell growth. These findings are somewhat contradictory to the usually observed aggressive clinical behavior in THRLBCL [30] and in a subset of cases of LP-DLBCL [10].

We also tried to model the prominent T cell- and macrophage-rich microenvironment of LP-DLBCL using the only available NLPHL cell line DEV [20, 39]. Despite DEV cells, as all cell lines, have acquired independence from their microenvironment, we made the observation that growth of DEV cells was hampered by the presence of T cells or monocytes, which could explain the reduced tumor cell content in LP-DLBCL next to a strong inflammatory background. Although we cannot exclude that reduced cell numbers of DEV cells after coculture were related to an increased rate of apoptosis, we could not prove this hypothesis in the data obtained from GEP. However, since GEP was performed on day 5, cell cycle arrest and growth inhibition may finally result in apoptosis at a later time point, suggested by a reduction of cell numbers in the growth curve clearly visible at day seven following a period of growth stagnation.

Taken together, the coculture conditions may represent environmental stress for DEV cells causing cell cycle arrest and growth inhibition via MYC downregulation. A lack of MYC expression was confirmed in the majority of LP-DLBCL tested, whereas in conventional DLBCL, around 50% of the cases show MYC expression in more than 30% of the tumor cells [40].

Since *CD274*/*PD-L1* was upregulated in DEV cells after coculture and in LP-DLBCL biopsies, checkpoint inhibitors could prevent the exhaustion of the relative high number of CD8-positive T cells in the microenvironment [41] and patients with LP-DLBCL could particularly benefit from such therapies. These results are in line with a previous study, in which expression of PD-L1 was noted in 91% of THRLBCL [42]. In contrast, conventional DLBCL presented PD-L1 expression only in rare cases in the present study, similar to the results reported in a previous study [43]. The observation that LP-DLBCL show a high content of CD8-positive T cells in most cases and expression of PD-L1 in the tumor cells is similar to the situation observed in other tumors like melanoma, which can basically be divided into tumors with and without recruitment of CD8-positive T cells [44]. The more mutated neoantigens are expressed by a tumor, the more immunogenic appear the tumor cells to the host immune system [45, 46]. Hence, several ways exist to evade immune system-mediated tumor destruction: One way is modulation of cytokine secretion which restricts T cell infiltration, another one is immune escape by MHC-downregulation and a third one, which is likely to be responsible for immune escape in LP-DLBCL, is functional inhibition of infiltrating CD8-positive T cells by PD-L1- or IDO-mediated anergy [47]. In addition to our observation of PD-L1 expression in the tumor cells of LP-DLBCL, IDO has also been reported to be expressed in macrophages and dendritic cells in the microenvironment of THRLBCL [35]. Therefore, both LP-DLBCL and THRLBCL present a potent host anti-lymphoma reaction, which is functionally impaired through immune modulation by the tumor cells.

## CONCLUSIONS

In conclusion, we observed a prominent host anti-lymphoma response in LP-DLBCL, which seems to be functionally impaired by PD-L1 expression of the tumor cells. Additional MYC downregulation in the tumor cells of LP-DLBCL possibly explains the low tumor cell content in these cases. Accelerating this ongoing anti-lymphoma reaction via immune checkpoint inhibitors may contribute to an improved survival of these patients.

## MATERIALS AND METHODS

### Gene expression analysis

Thirty-three primary cases of LP-DLBCL were collected in a previous study [9]. As control 11 DLBCL classified as GCB type and 9 DLBCL classified as Non-GCB type (Hans classifier [5]) were included. RNA was extracted using the RNeasy FFPE Microkit (Qiagen, Hilden, Germany). Since all cases showed partially degraded RNA profiles on an Agilent Bioanalyzer (Agilent, Waldbronn, Germany), a real time polymerase chain reaction (PCR) for GAPDH was performed after cDNA synthesis from 766 ng RNA with the High-capacity cDNA Reverse Transcription Kit (Applied Biosystems, Darmstadt, Germany). Cases with GAPDH Ct values above 30 proved to show insufficient quality when hybridized on gene expression arrays. Therefore, only cases with GAPDH values below 30 were further processed. Nine of the thirty-three LP-DLBCL qualified for the study according to their GAPDH values. Of these cases, 80 ng RNA were then transcribed using the NuGEN Ovation FFPE WTA System (NUGEN, Bemmel, The Netherlands). Gene arrays 1.0 (Affymetrix, Santa Clara, USA) were hybridized over night according to manufacturer's instructions. Data were analyzed as previously described [34]. Gene expression data are available through the GEO database (GSE84464). The local ethics committee of Frankfurt University Hospital agreed on the study (No. 39/14). For gene expression analysis of cultured cells, RNA was extracted using the RNeasy Mini Kit (Qiagen), amplified with the NUGEN Ovation Pico WTA System V2 (NUGEN) and hybridized onto Affymetrix Gene arrays 1.0. Gene expression data are available through the GEO database (GSE84688). Gene set characterization analysis was performed using the Genomatix Pathway System (Genomatix, Munich, Germany) at default parameters, listing all canonical pathways and biological terms with a significant enrichment of the provided input genes. Gene set enrichment analysis of the host response signature by Monti *et al.* [19] was performed according to http://software.broadinstitute.org/gsea/index.jsp [48, 49].

### Immunohistochemistry, western blot and quantitative real time PCR

Genes of interest identified by GEP were validated on protein level by immunohistochemistry as described previously [50]. Antibodies, providers and dilutions are listed in Supplementary Table S4. LP-DLBCL, DLBCL classified as GCB type or Non-GCB type (Hans classifier [5]), were stained on tissue microarray format. Stained slides were scanned using the Aperio Scan Scope XT scanner (Aperio, Leica Biosystems, Wetzlar, Germany) at 40x magnification. Positivity was evaluated using the Aperio Image Scope software and the Aperio Positive Pixel Count Algorithm quantifying the positive pixel count at default settings. At default settings, the intensity limits establish three intensity ranges for classifying and summing positive pixel values. In order to avoid a staining intensity bias due to fixation artifacts we considered for our analysis the sum of all positive pixels (including weakly positive, positive and strongly positive pixels). Pixels which are stained but do not fall into the positive color specification are considered as negative. These pixels are counted as well, so that the fraction of all positive pixels to total pixels is given.

For comparison of the fraction of positive pixels between the groups, the Mann-Whitney-Test or t-test was used, depending on the presence of a Gaussian distribution (Shapiro-Wilk-normality test). For Western blot analysis, standard conditions were applied. Antibodies, providers and dilutions are listed in Supplementary Table S4. Western blot images were acquired with a Fusion SL (Peqlab, Erlagen, Germany). Quantitative real time PCR (rtPCR) was performed on an ABI Prism 7900HT Fast Real-Time PCR System (Life Technologies, Darmstadt, Germany) using previously published primers for GAPDH [51] with Power SYBR Green PCR Master Mix (Applied Biosystems). The following intron-spanning primers for MYC were created: Forward 5′-GCAGCTGCTTAGACGCTGG-3′, Reverse 5′-CTCCTCGTCGCAGTAGAAATACG-3′. PCR products were sequenced, validating that the correct PCR product had been amplified. The cycling program consisted of 95°C for 10 min, followed by 40 cycles of 95°C for 15 s/60°C for 1 min. Expression of PD-L1 transcripts was performed using Taqman Universal PCR Master Mix and Gene Expression Assays for PD-L1 and GAPDH (all Applied Biosystems). Fold changes were calculated using the 2^−ΔΔCt^ method.

### Functional experiments

The NLPHL cell line DEV was analyzed for MHC class I and II expression by flow cytometry using specific fluorophore-conjugated antibodies, anti-HLA-ABC-APC (clone G462.6, BD Pharmingen, Heidelberg, Germany), anti-HLA-DR, DP, DQ-FITC (clone Tu39, BD Pharmingen). The HL cell line KM-H2 was used as positive control. The DEV cell line was cocultured with CD3-positive T cells or CD14-positive monocytes isolated from PBMCs of healthy donors by Ficoll density gradient centrifugation as described previously [22]. Proportions of PBMCs were subjected to negative isolation of T cells using the Pan T Cell Isolation Kit (Miltenyi Biotec, Bergisch-Gladbach, Germany) or positive selection of monocytes with CD14 Micro Beads (Miltenyi Biotec). Purities of at least 90% were obtained after magnetic cell separation (MACS) as it was determined by staining of the enriched fractions for CD3 (T cells) or CD14 (monocytes) and subsequent flow cytometry analysis.

Tumor cells were cocultured with reactive bystander cells in a ratio of 1:10 (2 × 10^5^ lymphoma cells and 2 × 10^6^ blood cells) in six well plates. Cell numbers were determined every two to three days by flow cytometry-based cell counting from cocultures seeded in triplicates, recognizing tumor cells by their unique pattern in forward and side scatter (FSC and SSC) and excluding T cells and monocytes by staining for surface molecules. The cultures were supplied with fresh medium every two to three days after cell counting and if required splitted 1:2 or 1:3 depending on the cell density. For gene expression analysis, tumor cells were purified from cocultures at day five of coculture by MACS using anti-CD4-, anti-CD8- and anti-CD14 Micro Beads (Miltenyi Biotec). Efficient removal of blood cell fractions was confirmed by flow cytometry analysis obtaining purities of > 94.0% after coculture experiments (Supplementary Figure S3).

## SUPPLEMENTARY FIGURES AND TABLES


