# Supplementary material for: A strong host response and lack of MYC expression are characteristic for diffuse large B cell lymphoma transformed from nodular lymphocyte predominant Hodgkin lymphoma
Source: Oncotarget. 2016 Sep 30;7(44):72197–210. doi: 10.18632/oncotarget.12363 (PMC5342154; doi:10.18632/oncotarget.12363)
Supplement: Supplementary file 1 [file oncotarget-07-72197-s001.pdf]

A strong host response and lack of MYC expression are characteristic for diffuse large B cell lymphoma transformed from nodular lymphocyte predominant Hodgkin lymphoma

SUPPLEMENTARY FIGURES AND TABLES

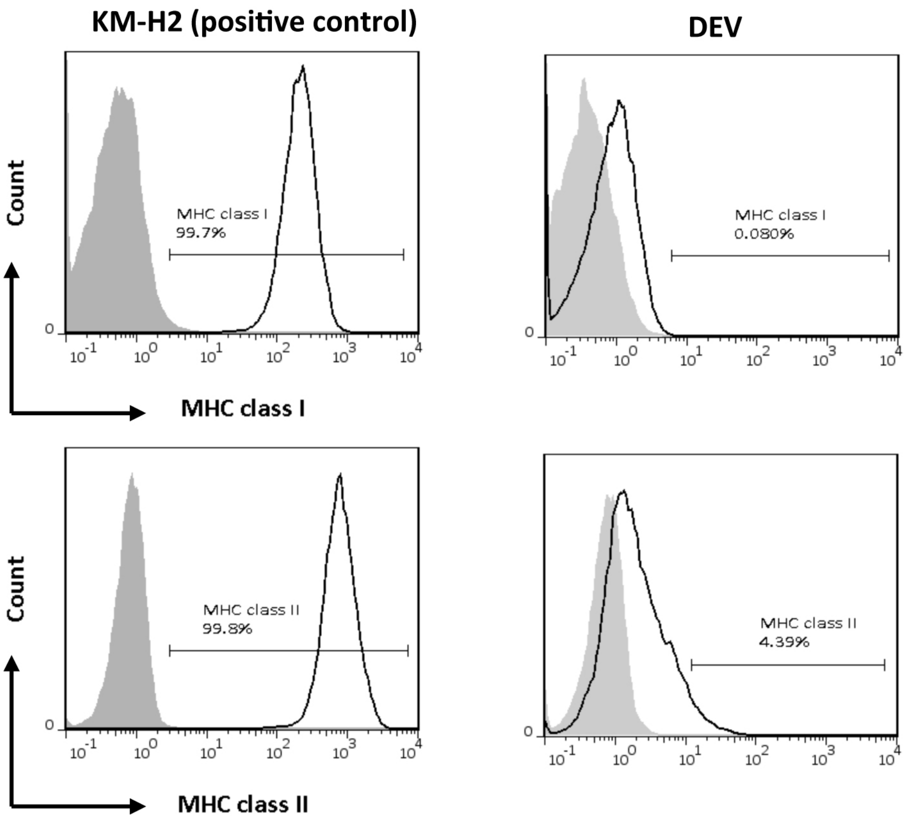

Supplementary Figure S1: MHC-Expression Histogram. Lack of MHC class I and -class II expression in DEV cells and presence on the surface of KM-H2 cells. Flow cytometry analysis of MHC class I (upper panel) and MHC class II (lower panel) in KM-H2 cHL cells and DEV cells showing unstained cells (grey histogram) compared to cells stained for MHC class I or -class II (white histogram with solid black line).

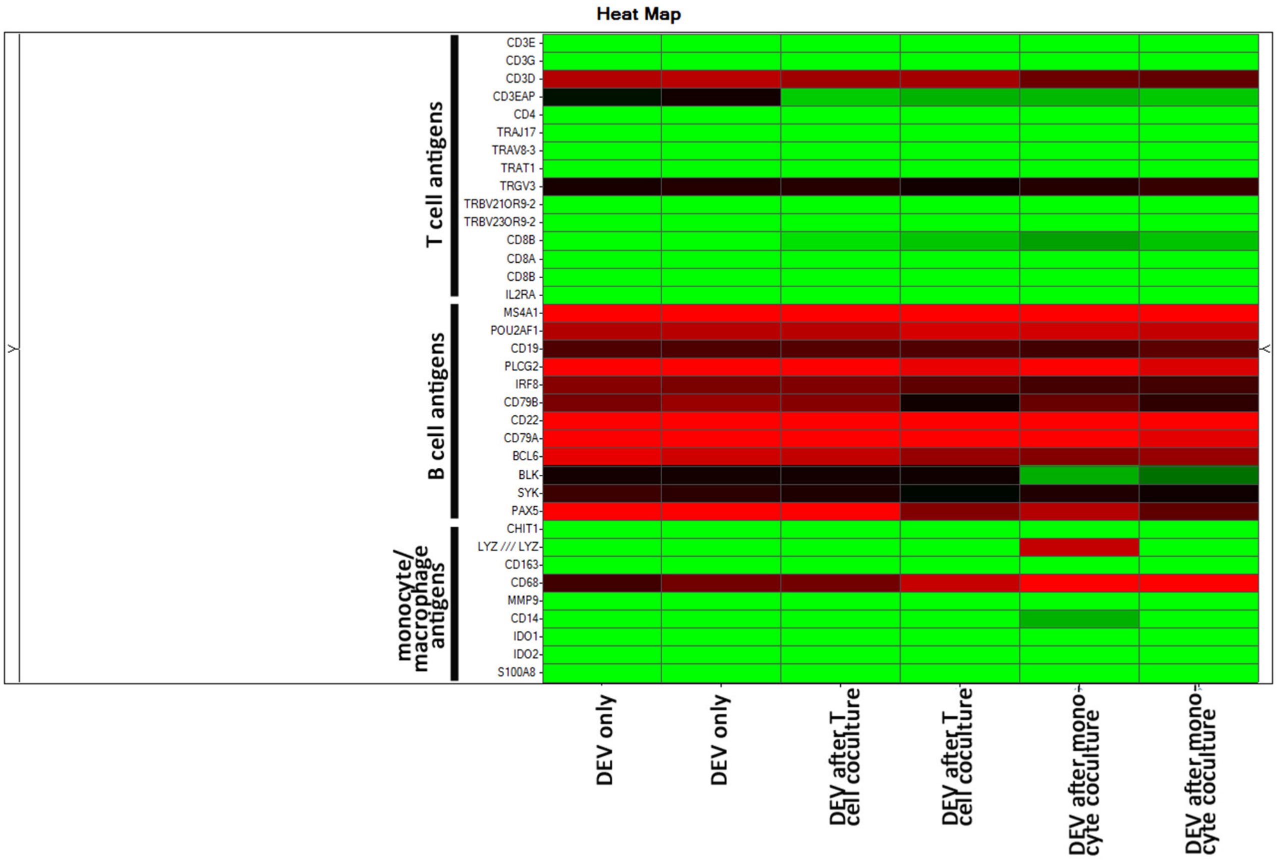

Supplementary Figure S2: Expression of T cell, B cell and monocyte/macrophage antigens in DEV cells and DEV cells after coculture with T cells and monocytes.

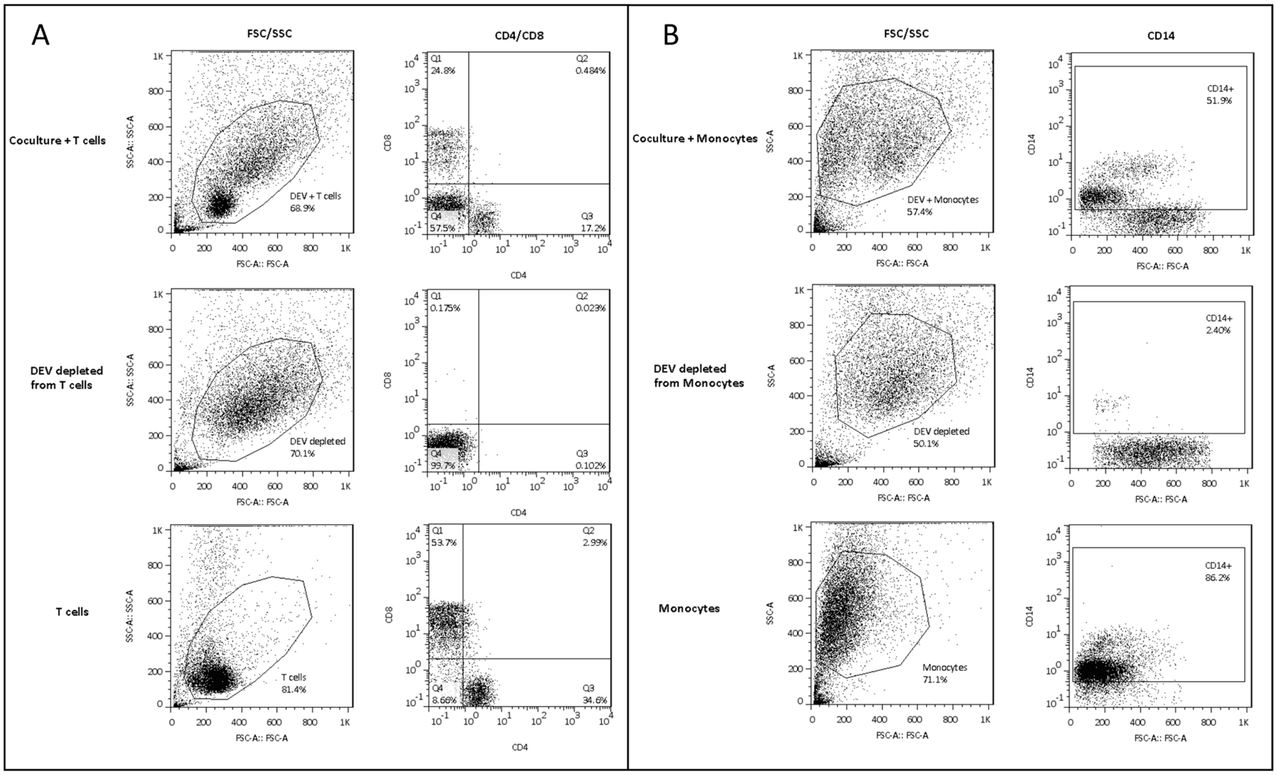

Supplementary Figure S3: DEV cells in coculture, after depletion of bystander cells, and depleted fraction. A. Coculture with T cells **B.** Coculture with monocytes. Efficient depletion of T cells (A) or monocytes (B) from cocultures with DEV cells by MACS. Flow cytometry analysis of CD4- and CD8-positive T cells or CD14-positive monocytes in coculture with DEV cells (upper panel), DEV cells purified from T cells or monocytes (middle panel) and T cell- or monocyte fractions depleted from cocultures (lower panel).

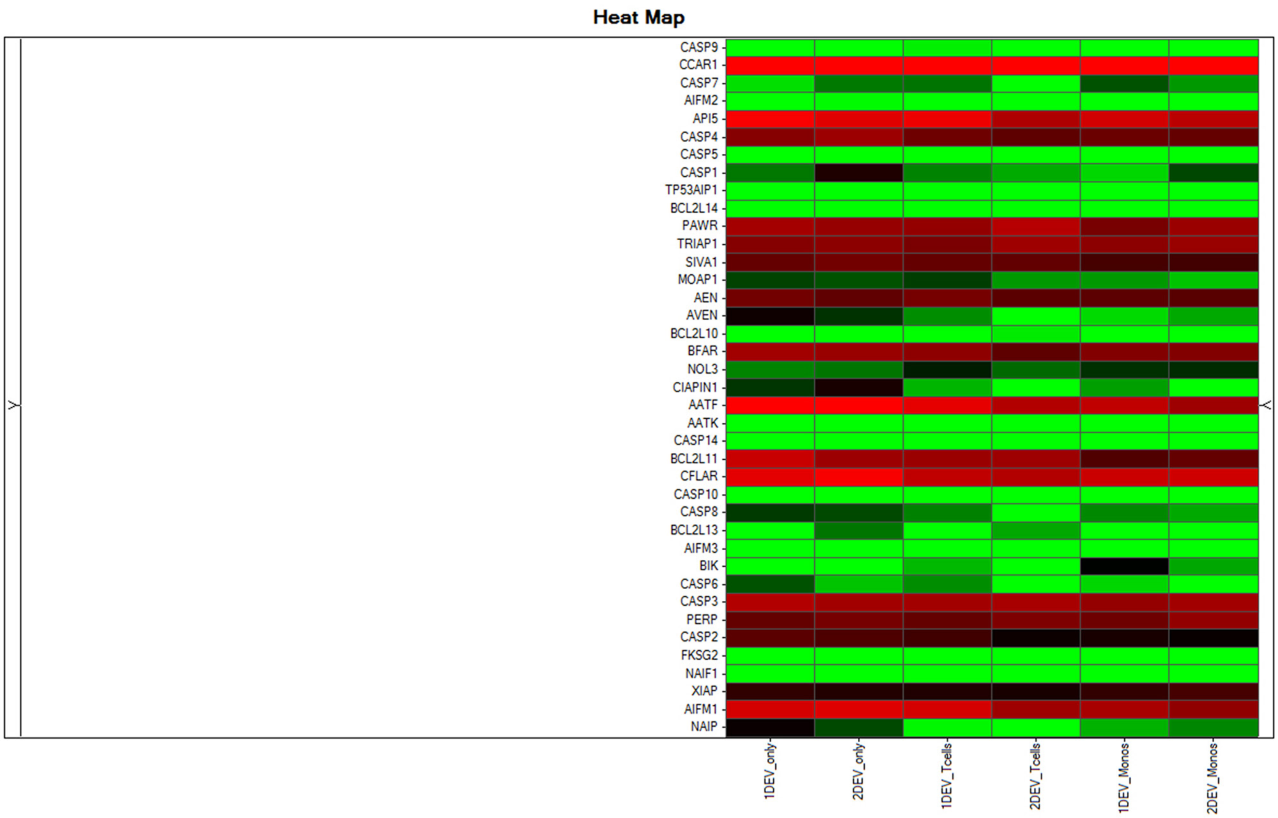

Supplementary Figure S4: Expression of apoptosis-related genes in DEV cells and DEV cells after coculture with T cells or monocytes.

Supplementary Table S1: Clinical features of LP-DLBCL and conventional DLBCL cases investigated by gene expression profiling

	Male gender (%)	Mean age (Range)	Simultaneous diagnosis of NLPHL + DLBCL (%)	Average time (years) to transformation in cases with sequential transformation: mean (Range)
LP-DLBCL (n=9)	9 (100%)	54 (23-78)	6 (67%)	10 (2-21)
GCB-DLBCL (n=11)	7 (64%)	55 (17-85)	-	-
Non-GCB DLBCL (n=9)	5 (56%)	64 (37-88)	-	-

Supplementary Table S2: Top 20 transcripts upregulated in DEV cells after coculture with T cells or monocytes

Fold change	p-value	FDR	Gene Symbol	Gene Description
5.3	0.000672643	0.044019241	*HSN2*	hereditary sensory neuropathy, type II
5.3	0.017829845	0.062068374	*RAB3A*	RAB3A, member RAS oncogene family
4.5	0.015681032	0.059258768	*FABP3*	fatty acid binding protein 3, muscle and heart (mammary-derived growth inhibitor)
3.7	0.01138228	0.056876836	*RRAGD*	Ras-related GTP binding D
3.6	0.008962452	0.054572689	*NPC2*	Niemann-Pick disease, type C2
3.5	0.003636499	0.052451694	*LY9*	lymphocyte antigen 9
3.4	0.033598833	0.080895651	*NR1D1*	nuclear receptor subfamily 1, group D, member 1
3.2	0.007026364	0.05271875	*FLJ38723*	hypothetical FLJ38723
3.1	0.011710716	0.057119147	*SNORD3A*	small nucleolar RNA, C/D box 3A
2.9	0.008426189	0.053305829	*ABHD4*	abhydrolase domain containing 4
2.9	0.001225376	0.049753082	*RGS1*	regulator of G-protein signaling 1
2.8	0.006621671	0.052451694	*CLEC2B*	C-type lectin domain family 2, member B
2.7	0.001833629	0.051676393	*GM2A*	GM2 ganglioside activator
2.7	0.016274548	0.05984063	*L1CAM*	L1 cell adhesion molecule
2.6	0.000891309	0.045529244	*TEC*	tec protein tyrosine kinase
2.6	0.014115387	0.059258768	*DDIT3*	DNA-damage-inducible transcript 3
2.6	0.002187248	0.051676393	*C3orf37*	chromosome 3 open reading frame 37
2.6	0.019910142	0.06391666	*LCP2*	lymphocyte cytosolic protein 2 (SH2 domain containing leukocyte protein of 76kDa)
2.6	0.009492806	0.055012047	*CDK2*	cyclin-dependent kinase 2
2.6	0.025536307	0.070811641	*MGC16384*	hypothetical LOC114130

FDR, false discovery rate

Supplementary Table S3: Top 20 transcripts downregulated in DEV cells after coculture with T cells or monocytes

Fold change	p-value	FDR	Gene Symbol	Gene Description
-8.4	0.012608853	0.058327178	*MYC*	v-myc myelocytomatosis viral oncogene homolog (avian)
-5.9	0.000478582	0.044019241	*MIR155*	microRNA 155
-4.7	0.005841808	0.052451694	*P2RY14*	purinergic receptor P2Y, G-protein coupled, 14
-4.4	0.013080596	0.059258768	*ACTG2*	actin, gamma 2, smooth muscle, enteric
-4.0	0.009958711	0.0551286	*TLR10*	toll-like receptor 10
-3.8	0.025376177	0.070811641	*UGT2B7*	UDP glucuronosyltransferase 2 family, polypeptide B7
-3.4	0.013699511	0.059258768	*FKBP5*	FK506 binding protein 5
-3.4	0.005210678	0.052451694	*FAM111B*	family with sequence similarity 111, member B
-3.4	0.007927227	0.05271875	*HNRNPM*	heterogeneous nuclear ribonucleoprotein M
-3.4	0.037738098	0.084978594	*RUVBL1*	RuvB-like 1 (E. coli)
-3.2	0.000124404	0.030982853	*MAT2A*	methionine adenosyltransferase II, alpha
-3.1	0.000755921	0.044019241	*NOP58*	NOP58 ribonucleoprotein homolog (yeast)
-3.0	0.014662792	0.059258768	*FUS*	fused in sarcoma
-3.0	0.006827164	0.052451694	*SFRS7*	splicing factor, arginine/serine-rich 7, 35kDa
-2.9	0.02628985	0.071788205	*AIM2*	absent in melanoma 2
-2.9	0.026732974	0.072258332	*NOP16*	NOP16 nucleolar protein homolog (yeast)
-2.9	0.002053548	0.051676393	*DKFZp434H1419*	hypothetical protein DKFZp434H1419
-2.8	0.001905462	0.051676393	*SAMD9L*	sterile alpha motif domain containing 9-like
-2.8	0.004897793	0.052451694	*EBNA1BP2*	EBNA1 binding protein 2
-2.7	0.002617768	0.051676393	*TIPIN*	TIMELESS interacting protein

FDR, false discovery rate

Supplementary Table S4: Antibodies, providers and dilutions used for immunohistochemistry (IHC) or Western Blot (WB)

Application	Antibody	Clone	Provider	Dilution	Order Number
IHC	CD3	Rabbit polyclonal	Dako, Glostrup, Denmark	1:200	A0452
IHC	CD20	L26	Dako	1:1000	M0755
IHC	p-STAT1	ST1P-11A5	Thermo Fisher, Rockford, IL, USA	1:100	333400
IHC	C1QA	Rabbit polyclonal	Sigma Aldrich, Taufkirchen, Germany	1:100	HPA002350
IHC	CD163	MRQ-26	Cell Marque, Rocklin, CA, USA	1:100	16311-15
IHC	CD4	4B12	Dako	Ready to use	IR649
IHC	CD8	C8/144B	Dako	1:100	M7103
IHC/WB	CD274/PD-L1	E1L3N	Cell Signaling Technology, Leiden, The Netherlands	1:50	#13684
IHC	MYC	Y69	Abcam, Cambridge, UK	1:100	Ab32072
WB	MYC	D84C12	Cell Signaling	1:1000	#5605S
WB	ACTB	AC-74	Sigma-Aldrich, St Louis, USA	1:20000	A2228
WB	Anti-rabbit-HRP	Goat polyclonal	Dako	1:2000	P0448
WB	Anti-mouse-HRP	Goat polyclonal	Dako	1:10000	P0447
